# A meta-analysis on the prevalence, associated factors and diagnostic methods of mental stress induced myocardial ischemia

**DOI:** 10.1186/s12967-020-02383-z

**Published:** 2020-05-29

**Authors:** Lijun Zhang, Yanping Bao, Xi Wang, Yuxin Zhou, Shuhui Tao, Wan Xu, Meiyan Liu

**Affiliations:** 1 Cardiology Department, Beijing Anzhen Hospital, Capital Medical University, No. 2 Anzhen Road, Chaoyang District, Beijing, 100029 China; 2 National Institute on Drug Dependence, Peking University, No. 38, Xueyuan Road, Haidian District, Beijing, 10091 China; 3 Department of Anatomy and Neurobiology, Boston University, Boston, USA; 4 School of Basic Medical Sciences, Henan University, Kaifeng, China

**Keywords:** Mental stress, Myocardial ischemia, Diagnostic method, Meta-regression

## Abstract

**Background:**

The high prevalence of mental stress induced myocardial ischemia (MSIMI) causes double risk of adverse cardiac events in patients with MSIMI. However, multiple types of mental stress, diagnostic techniques, and diagnostic measurements may increase the complexity and heterogeneity in the assessment of MSIMI. Therefore, we performed this meta-analysis to assess the prevalence, associated factors, and diagnostic methods of MSIMI.

**Methods:**

We systematically searched PubMed, EMBACE, Web of Science, CNKI, Wanfang through 1 Feb 2020 in English and Chinese. Review Manager (RevMan) Version 5.3 and Stata 12.0 were used for data analyses.

**Results:**

Twenty articles were enrolled. The pooled estimates for the prevalence of MSIMI in CAD patients was 32%. Potential associated factors of MSIMI involved history of post myocardial infarction (MI), or coronary artery bypass graft (CABG) (RR: 1.29, 95% CI 1.00–1.66, P = 0.05; RR: 1.59, 95% CI 1.00–2.52, P = 0.05). Evidence supported that diagnostic methods could influence the prevalence of MSIMI. Significant differences of MSIMI prevalence were found in different types of mental stress (Public Speaking: 22%; Mental arithmetic: 26%; Anger recall: 34%; Two types: 37%; Three or more than three types: 43%, P = 0.02), diagnostic techniques (SPECT: 26%; RNV: 38%; ECG: 16%; Echocardiography: 41%; Two types: 43%, P < 0.0001), and diagnostic measurements (LVEF decrease: 19%; WMA: 51%; ST depression: 16%; MPD: 26%; Two or more than two measurements: 45%, P < 0.00001). Moreover, univariate meta-regression demonstrated that MSIMI was linked with mental stress (exp(b): 1.0508, SE: 0.0201, P: 0.018).

**Conclusions:**

This meta-analysis implicated that patients with diabetes, post MI or CABG might be more vulnerable to MSIMI. However, the prevalence of MSIMI could be influenced by diagnostic methods, especially the adopted types of mental stress, diagnostic techniques and measurements. Therefore, it is necessary to formulate a standard diagnostic method for MSIMI, which should be adequate, assessable, and affordable worldwide.

*Registration* PROSPERO. Online Protocol: CRD42020162822.

## Background

Cardiovascular diseases (CVD) have been threatening human’s life for a long time all around the world, which could lead to 23.3 million deaths by 2030 according to the report from World Health Organization [1]. In China, there are more than 290 million patients with established cardiovascular diseases, and the mortality keeps rising [2]. Tens of billions of dollars have been spent in CVD management with limited effect. Despite traditional risk factors such as smoke, hypertension, hyperlipidemia, and diabetes [3], increasing evidence identified mental stress as a crucial risk factor in the development and progression of CVD [4]. Researchers discovered that mental stress induced in the laboratory (e.g. mental arithmetic, public speaking, et al.) contributed to myocardial ischemia, which could be assessed by echocardiography, electrocardiogram, or SPECT (single photonemission computed tomography) [5–7]. The prevalence of mental stress induced myocardial ischemia (MSIMI) ranges from 50 to 70% in patients with coronary artery diseases (CAD) [8]. Compared with patients without MSIMI, patients with MSIMI have double risk of adverse cardiac events [8].

However, the mechanisms of MSIMI remain uncertain. Previous studies showed that emotional status such as the trait and state of anger [5], anxiety [6], depression [7] could have great impact. Potential mechanisms may involve inflammatory responses, cortisol responses, fibrinogen responses, coagulation system, hypothalamic pituitary adrenal (HPA) [9, 10]. Hammadah et al. [11] linked cardiac biomarker with MSIMI, presenting that patients with MSIMI had higher level of resting cTnI. However, various factors such as sex, race, disease history, and drug history, and multiple types of mental stress, diagnostic techniques, and diagnostic measurements increase the complexity and heterogeneity in the assessment of MSIMI. Therefore, we performed this meta-analysis and meta-regression in an effort to explore the potential mediators of MSIMI.

## Methods

### Article selection strategy

This meta-analysis had been registered in PROSPERO (CRD42020162822). We conducted the present meta-analysis by searching PubMed, Embase, Web of science, China National Knowledge Infrastructure (CNKI), and Wanfang website through 1 Feb 2020, with key words “mental stress”, “psychological stress”, “myocardial ischemia”, “mental stress ischemia”, “mental stress induced myocardial ischemia”, “MSIMI”.

Inclusion criteria: (1) Prospective cohort study or cross-sectional study; (2) English or Chinese language; (3) Patients with coronary artery disease; (4) Full articles were able to be found; (5) The data were eligible to be extracted; (6) Articles with high or medium quality.

Exclusion criteria: (1) Articles with repeated data from the same study project; (2) Mental stress tasks followed exercise stress at the same day, which might implicated that myocardial ischemia could be induced by exercise stress rather than mental stress.

### Article selection steps

Three authors focused on selecting the proper articles in nearly 1 month. There were four steps in article selection and data extraction. First, the authors read the titles and excluded those unsuitable; Second, they read the abstracts and included those articles in the scope of our research; Third, they downloaded the articles with full text through the internet or our country library; Fourth, they read all articles, extracted necessary data for this study, and excluded articles without qualified data. Agreement must be reached among three authors to process the data.

### Quality assessment

The quality of the cross-sectional studies was assessed by Crombie tool [12]. According to the scores, the article was classified into Grade A (6.0–7.0 points), Grade B (4.0–5.5 points), Grade C (< 4 points). Articles with Grade A were regarded as high quality, Grade B as moderate quality, and Grade C as low quality. The quality of the prospective cohort studies was assessed by Newcastle-Ottawa Scale (NOS) [13]. Articles with seven to nine stars were estimated as high quality, five to six stars as medium quality, and zero to four stars as poor quality. Articles with high or medium quality were included in the present study (Table 1).

**Table 1:** The characteristics of the articles

No.	First author	Publication date	Country	Study type (follow-up)	mental stress	Diagnostic technique	Diagnostic criteria	Sex	Age (years)	Total sample	MSIMI	Scores of Crombie/NOS	Article quality
1	Akinboboye	2005	USA	Cross-sectional study (No)	Anger recall Mental arithmetic	SPECT	MPD	Male Female	62.82 ± 8.71^a^ 61.63 ± 7.99^b^	58	17	5	Moderate
2	Babyak	2010	USA	Prospective cohort study (5.9 years)	Public speaking Mirror trace	RNV	LVEF decrease	Male Female	62.5 (55.8, 71.2)^a^ 60.0 (51.2, 69.0)^b^ 62.0 (55.0, 70.0)^c^	138	26	9 stars	High
3	Burg	2009	USA	Cross-sectional study (No)	Ager recall	SPECT	MPD	Male Female	66.2 ± 9.7^a^ 64.9 ± 6.9^b^ 65.9 ± 8.9^c^	68	22	6	High
4	Carels	1999	USA	Cross-sectional study (No)	Mental arithmetic Public speaking Mirror tracing Reading Type A structured interview	RNV Ambulatory ECG	WMA ST depression	Male Female	58.5 ± 8.4^c^	136	79	6	High
5	Hammadah	2017	USA	Cross-sectional study (No)	Public speaking	SPECT	MPD	Male Female	62.9 ± 9.1^c^	660	106	6	High
6	Hassan	2007	USA	Cross-sectional study (No)	Public speaking	SPECT	MPD	Male Female	64 ± 9^c^	182	38	6	High
7	Hassan	2009	USA	Cross-sectional study (No)	Public speaking	SPECT	MPD	Male Female	64 (mean)^c^	211	34	6	High
8	Jiang	2013	USA	Cross-sectional study (No)	Mental arithmetic Mirror trace Anger recall	Echocardiography	LVEF decrease WMA	Male Female	63.35 ± 10.33^a^ 63.63 ± 10.73^b^ 63.81 ± 10.48^c^	307	134	6	High
9	Krantz	1991	USA	Cross-sectional study (No)	Mental arithmetic Stroop color-word task Public speaking Reading	RNV	WMA	Male Female	59.1 ± 11.3^a^ 60.2 ± 11.4^b^	39	23	5	Moderate
10	Krantz	1999	USA	Prospective cohort study (3.5 years)	Mental arithmetic Public speaking	Echocardiography RNV	WMA	Male Female	58 ± 10^c^	79	28	5	Moderate
11	Liu	2019	China	Cross-sectional study (No)	Mental arithmetic	Echocardiography	LVEF decrease	Male Female	60.2 ± 9.7^a^ 59.8 ± 10.2^b^ 60.1 ± 9.8^c^	82	16	6	High
12	Shah	2006	USA	Cross-sectional study (No)	Anger recall	SPECT Echocardiography	MPD WMA	Male Female	67.2 (mean)^a^ 66.0 (mean)^b^	83	30	6	high
13	Sheps	2002	USA	Prospective cohort study (4.3–6.0 years)	Stroop color-word task Public speaking	RNV ECG/Ambulatory ECG	ST depression LVEF decrease WMA	Male Female	62.6 ± 8.1^a^ 62.8 ± 9.1^c^	182	77	9 stars	High
14	Soufer	2016	USA	Cross-sectional study (No)	Mental arithmetic	SPECT	MPD	Male Female	65.6 ± 9.0^c^	161	64	6	High
15	Specchia	1984	Italy	Cross-sectional study (No)	Mental arithmetic	ECG	ST depression	Male Female	50.5 ± 7^c^	111	20	6	High
16	Stepanovic	2012	Serbia	Cross-sectional study (No)	Mental arithmetic Anger recall	Echocardiography	WMA	Male Female	52 ± 8^c^	79	48	6	High
17	Vaccarino	2014	USA	Cross-sectional study (No)	Public speaking	SPECT	MPD	Male Female	50 (mean)^c^	93	36	6	High
18	Vaccarino	2018	USA	Cross-sectional study (No)	Public speaking	SPECT	MPD	Male Female	50.5 (mean)^c^	306	50	6	High
19	Wong	1997	Australia	Cross-sectional study (No)	Mental arithmetic Stroop color-word task Reading Public speaking Competitivecomputer game	ECG	ST depression	Male Female	61 ± 9^c^	35	4	5	Moderate
20	York	2007	USA	Cross-sectional study (No)	Public speaking	SPECT	MPD	Male Female	63 ± 8.58^c^	154	50	6	High

*MPD* myocardial perfusion defects, *WMA* wall motion abnormality, *LVEF* left ventricle ejection fraction, *RNV* radionuclide ventriculography, *ECG* electrocardiography, *MPD* myocardial perfusion defects, *SPECT* single photon emission computed tomography

^a^The average age of patients without MSIMI

^b^Thea average age of patients with MSIMI

^c^The average age of total patients

### Data extraction

The data were extracted by two researchers separately and reached agreement after consultation. The following data were extracted: first author; publication date; country; total sample size; the sample of patients with MSIMI; study type; mental stress; diagnostic methods; scores of Crombie/NOS; article quality. All the data were presented in Table 1.

### Diagnostic methods of MSIMI

1. Mental stress: Participants received one or more than one type of mental stress for 5 min, involving the most common types: mental arithmetic, public speaking, mirror trace, Stroop color word task, and several other uncommon types of mental stress (such as reading).
2. Diagnostic techniques: Several techniques were adapted to evaluate cardiac function before and after participants went through mental stress, such as electrocardiogram (ECG), echocardiography, single photon emission computed tomography (SPECT), ventricular function monitor, radionuclide ventriculography (RNV).
3. Diagnostic criteria: Researchers have developed 4 criteria to diagnose MSIMI, including left ventricular ejection fraction (LVEF) decrease ≥ 5% or 8%, new or worsen wall motion abnormality, myocardial perfusion defect, ST depression ≥ 0.1 mV. Any of the four criteria could be adequate to diagnose MSIMI.

More details about diagnostic methods were shown in Table 2.

**Table 2:** The details of diagnostic methods

Diagnostic methods	Types	Details
Mental stress	Mental arithmetic	Participants were required to complete a series of mathematical calculation, for instance, to subtract 7 from a 4-digit number in 5 min as quickly as possible, at the same time, they would receive encouragement or discouragement from the investigators
	Public speaking	Participants were asked to give a speech on a topic given by the investigators, and they had 2 min to prepare and 3 min to deliver the speech. They were told that their speech would be recorded and evaluated by the investigators
	Mirror trace	Participants were instructed to outline the shape of a star from its reflection in a mirror
	Stroop color word task	Participants were showing a series of slides which displaying the written word of a non-matching color (e.g. the word green in blue color)
	Anger recall	Participants were asked to recall a recent annoying event which made them feel angry, upset, irritated, frustrated, then described the situation and feeling to the investigators in details
	Reading	Participants were asked to read a passage given by the investigators, such as neutral passage, in front of the investigators
	Type A structured interview	Participants underwent a standard videotaped interview to assess type A behavior which might last 20 min
	Competitive computer game	Participants were asked to play a kind of computer game, which might elicit threat, uncertainty, and avoidance.
Diagnostic techniques	SPECT	[99mTc] sestamibi SPECT was used to acquire myocardial perfusion imaging at rest and during mental stress
	RNV	R-wave synchronized, multiple-gated RNV was conducted with a gamma camera positioned in the left anterior oblique angle, to acquire LVEF and left ventricular wall motion
	ECG/Ambulatory ECG	12 lead ECG or an ambulatory ECG was used for recording ST segments
	Echocardiography	Two dimensional echocardiography was used to assess regional wall motion and LVEF
Diagnostic measurements	LVEF decrease	A reduction of LVEF at least 5% or 8% during mental stress compared with rest LVEF was considered to exhibit MSIMI
	WMA	New or worsened wall motion abnormalities during mental stress when compared with rest
	ST depression	At least 1 mm ST segment depression by ECG or ambulatory ECG
	MPD	A 17-segment model was used to assess the myocardial perfusion defects comparing rest and mental stress images, The following considerations could be regarded as MSIMI: a new myocardial perfusion defect with a score of 2 in any segment, or worsening of a preexisting impairment of at least 2 points in a single segment, or worsening of at least 1 point in 2 or more contiguous segments

*MPD* myocardial perfusion defects, *WMA* wall motion abnormality, *LVEF* left ventricle ejection fraction, *RNV* radionuclide ventriculography, *ECG* electrocardiography, *MPD* myocardial perfusion defects, *SPECT* single photon emission computed tomography, *MSIMI* mental stress induced myocardial ischemia

### Statistical analysis

Review Manager (RevMan) Version 5.3 and Stata 12.0 were adopted for data analyses. Cochran’s Q-test [14] and I^2^ statistic [15] were used for heterogeneity. Pooled effect size was analyzed by random-effects model or fixed-effects model according to the level of heterogeneity. Random-effects model was established for significant heterogeneity (P < 0.10 or I^2^ > 50%), while fixed-effects model was used for non-significant heterogeneity (P > 0.10 or I^2^ < 50%). Meta-Regression and subgroup analysis were applied for seeking heterogeneity sources. Sensitivity analyses were performed via excluding studies one at a time [16]. Publication bias was estimated by funnel plot and Begg’ test [17]. P values were two-sided, and P < 0.05 was considered statistically significant.

## Results

### Prevalence of MSIMI in patients with CAD

**Fig. 1:**
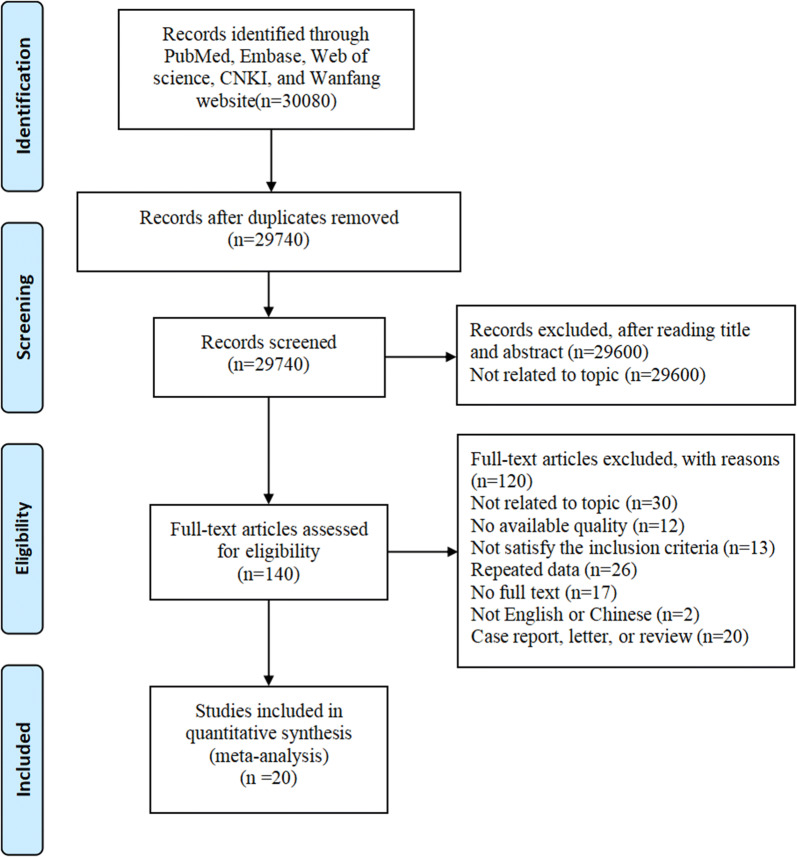
The flow diagram of meta-analysis on mental stress induced myocardial ischemia; CNKI: China National Knowledge Infrastructure

For this meta-analysis, a total number of 30,080 publications were found from the databases. After removing duplication and articles unrelated to the topic, 20 eligible articles were finally selected [18–37]. Sixteen studies came from USA, and other four came from China, Serbia, Italy, Australia respectively. The flow chart was presented in Fig. 1. This meta-analysis enrolled 3164 patients with CAD, including 902 patients with MSIMI, and 2262 patients without MSIMI. The characteristics of all the articles were presented in Table 1 (Fig. 1, Table 1).

**Fig. 2:**
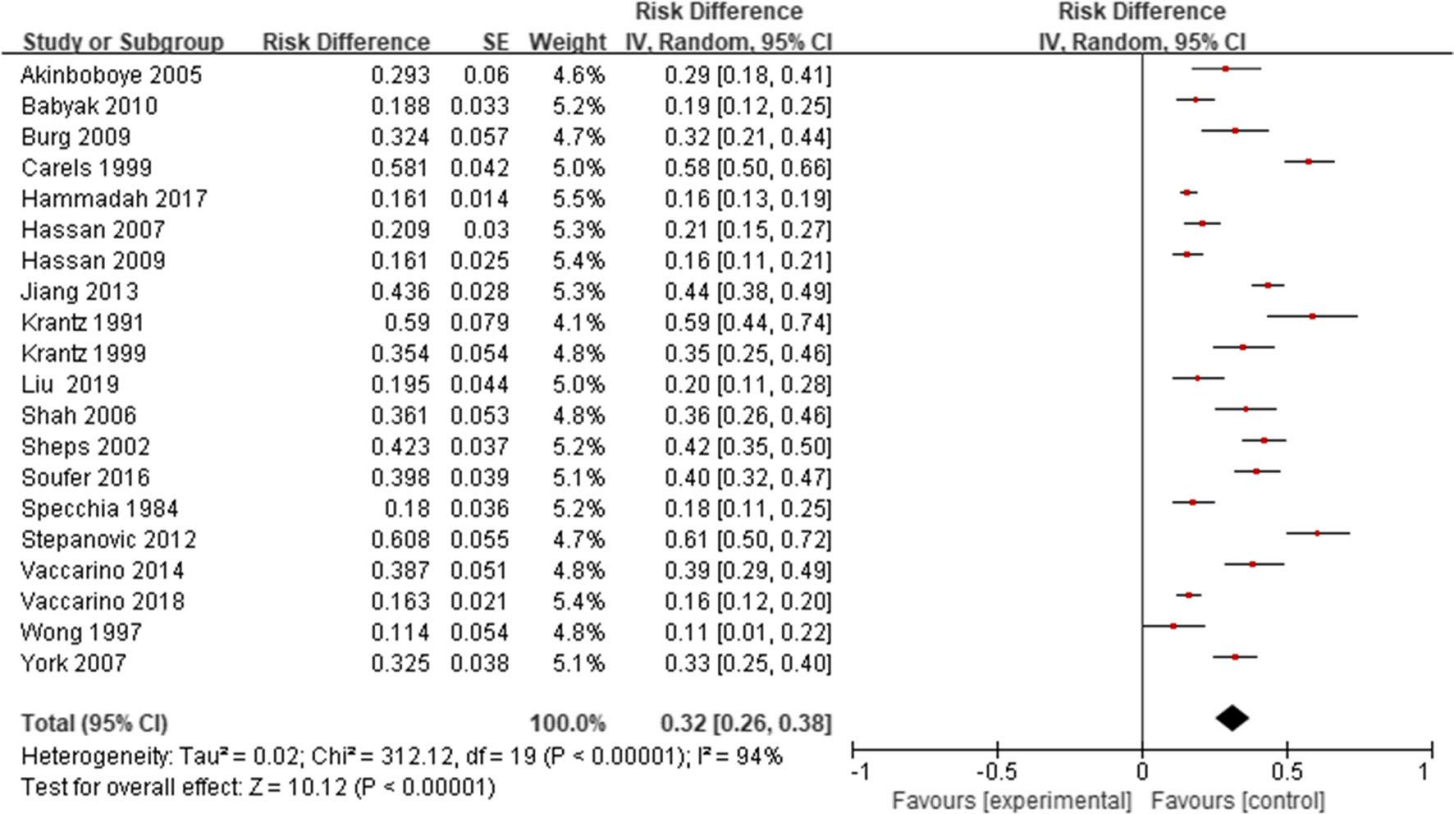
The prevalence of MSIMI in CAD patients

Of the 20 studies, the prevalence of MSIMI in CAD patients ranges from 11 to 61%. In this meta-analysis, the pooled estimate for the prevalence of MSIMI in CAD patients is 32% (95% CI 0.26, 0.38) (Fig. 2). We performed subgroup analyses of MSIMI prevalence, according to sex (Female 30%, Male 31%), race (White 40%, non-white 47%), smoking (Smoke+ 34%, Smoke− 31%), disease history (Hypertension+ 34%, Hypertension− 30%, Hyperlipidemia+ 36%, Hyperlipidemia− 29%, Diabetes+ 38%, Diabetes− 31%, Depression+ 56%, Depression− 31%, Post MI+ 38%, Post MI− 32%, PTCA+ 32%, PTCA− 34%, CABG+ 37%, CABG− 30%), and drug history (Aspirin+ 33%, Aspirin− 32%, Other antiplatelets+ 33%, Other antiplatelets− 32%, ACEI+ 34%, ACEI− 33%, ARB+ 35%, ARB− 29%, β-block+ 31%, β-block− 30%, CCB+ 32%, CCB− 34%, Statins+ 31%, Statins− 19%) (Table 3).

**Table 3:** Prevalence of subgroups analyses

Subgroups	No. of studies	Total	MSIMI	Pooled prevalence(%)	95%CI	Effect Model	Heterogeneity	Test for overall effect
Basic characteristics								
Female	11	612	162	30	0.21, 0.39	Random	Tau^2^ = 0.02; Chi^2^ = 55.51, df = 10 (P < 0.00001); I^2^ = 82%	Z = 6.66 (P < 0.00001)
Male	11	1519	420	31	0.22, 0.40	Random	Tau^2^ = 0.02; Chi^2^ = 148.60, df = 10 (P < 0.00001); I^2^ = 93%	Z = 7.07 (P < 0.00001)
White	3	440	175	40	0.35, 0.44	Fix	Chi^2^ = 1.37, df = 2 (P = 0.50); I^2^ = 0%	Z = 17.02 (P < 0.00001)
Other races	3	96	45	47	0.37, 0.57	Fix	Chi^2^ = 2.94, df = 2 (P = 0.23); I^2^ = 32%	Z = 0.54 (P = 0.59)
Smoke+	8	902	256	34	0.21, 0.46	Random	Tau^2^ = 0.03; Chi^2^ = 103.54, df = 7 (P < 0.00001); I^2^ = 93%	Z = 5.43 (P < 0.00001)
Smoke−	8	676	190	31	0.20, 0.42	Random	Tau^2^ = 0.02; Chi^2^ = 65.09, df = 7 (P < 0.00001); I^2^ = 89%	Z = 5.48 (P < 0.00001)
Hypertension+	8	1211	355	34	0.23, 0.44	Random	Tau^2^ = 0.02; Chi^2^ = 102.11, df = 7 (P < 0.00001); I^2^ = 93%	Z = 6.25 (P < 0.00001)
Hypertension−	8	367	91	30	0.18, 0.43	Random	Tau^2^ = 0.03; Chi^2^ = 52.54, df = 7 (P < 0.00001); I^2^ = 87%	Z = 4.72 (P < 0.00001)
Hyperlipidemia+	8	1255	381	36	0.24, 0.47	Random	Tau^2^ = 0.03; Chi^2^ = 135.94, df = 7 (P < 0.00001); I^2^ = 95%	Z = 6.09 (P < 0.00001)
Hyperlipidemia−	8	185	39	29	0.17, 0.40	Random	Tau^2^ = 0.01; Chi^2^ = 15.92, df = 6 (P = 0.01); I^2^ = 62%	Z = 4.86 (P < 0.00001)
Diabetes+	8	466	142	38	0.25, 0.52	Random	Tau^2^ = 0.03; Chi^2^ = 63.60, df = 7 (P < 0.00001); I^2^ = 89%	Z = 5.54 (P < 0.00001)
Diabetes−	8	1112	304	31	0.20, 0.42	Random	Tau^2^ = 0.02; Chi^2^ = 113.93, df = 7 (P < 0.00001); I^2^ = 94%	Z = 5.58 (P < 0.00001)
Depression+	2	71	28	56	0.15, 2.08	Random	Tau^2^ = 0.75; Chi^2^ = 5.93, df = 1 (P = 0.01); I^2^ = 83%	Z = 0.87 (P = 0.39)
Depression−	2	318	122	31	0.07, 0.54	Random	Tau^2^ = 0.03; Chi^2^ = 15.40, df = 1 (P < 0.0001); I^2^ = 94%	Z = 2.58 (P = 0.010)
Post MI+	5	585	189	38	0.21, 0.55	Random	Tau^2^ = 0.03; Chi^2^ = 78.29, df = 4 (P < 0.00001); I^2^ = 95%	Z = 4.43 (P < 0.00001)
Post MI−	5	760	189	32	0.17, 0.46	Random	Tau^2^ = 0.02; Chi^2^ = 62.36, df = 4 (P < 0.00001); I^2^ = 94%	Z = 4.36 (P < 0.0001)
PTCA+	3	633	168	32	0.11, 0.53	Random	Tau^2^ = 0.03; Chi^2^ = 59.22, df = 2 (P < 0.00001); I^2^ = 97%	Z = 2.97 (P = 0.003)
PTCA−	3	495	136	34	0.14, 0.54	Random	Tau^2^ = 0.03; Chi2 = 37.29, df = 2 (P < 0.00001); I^2^ = 95%	Z = 3.40 (P = 0.0007)
CABG+	3	432	148	37	0.21, 0.54	Random	Tau^2^ = 0.02; Chi^2^ = 22.92, df = 2 (P < 0.00001); I^2^ = 91%	Z = 4.57 (P < 0.00001)
CABG−	3	696	156	30	0.08, 0.52	Random	Tau^2^ = 0.04; Chi^2^ = 70.62, df = 2 (P < 0.00001); I^2^ = 97%	Z = 2.67 (P = 0.008)
Aspirin+	5	1081	298	33	0.18, 0.48	Random	Tau^2^ = 0.03; Chi^2^ = 91.34, df = 4 (P < 0.00001); I^2^ = 96%	Z = 4.34 (P < 0.0001)
Aspirin−	5	198	58	32	0.20, 0.45	Random	Tau^2^ = 0.01; Chi^2^ = 12.74, df = 4 (P = 0.01); I^2^ = 69%	Z = 5.08 (P < 0.00001)
Other antiplatelets+	3	396	117	33	0.11, 0.55	Random	Tau^2^ = 0.04; Chi^2^ = 38.94, df = 2 (P < 0.00001); I^2^ = 95%	Z = 2.96 (P = 0.003)
Other antiplatelets−	3	732	187	32	0.12, 0.51	Random	Tau^2^ = 0.03; Chi^2^ = 59.37, df = 2 (P < 0.00001); I^2^ = 97%	Z = 3.19 (P = 0.001)
ACEI+	5	658	201	34	0.21, 0.47	Random	Tau^2^ = 0.02; Chi^2^ = 42.57, df = 4 (P < 0.00001); I^2^ = 91%	Z = 5.24 (P < 0.00001)
ACEI−	5	621	155	33	0.17, 0.49	Random	Tau^2^ = 0.03; Chi^2^ = 58.21, df = 4 (P < 0.00001); I^2^ = 93%	Z = 4.03 (P < 0.0001)
ARB+	2	149	38	35	− 0.08, 0.78	Random	Tau^2^ = 0.09; Chi^2^ = 26.62, df = 1 (P < 0.00001); I^2^ = 96%	Z = 1.61 (P = 0.11)
ARB−	2	818	202	29	0.04, 0.54	Random	Tau^2^ = 0.03; Chi^2^ = 54.50, df = 1 (P < 0.00001); I^2^ = 98%	Z = 2.30 (P = 0.02)
β-block+	6	1086	301	31	0.19, 0.42	Random	Tau^2^ = 0.02; Chi^2^ = 85.31, df = 5 (P < 0.00001); I^2^ = 94%	Z = 5.01 (P < 0.00001)
β-block−	6	331	81	30	0.19, 0.41	Random	Tau^2^ = 0.01; Chi^2^ = 20.32, df = 5 (P = 0.001); I^2^ = 75%	Z = 5.31 (P < 0.00001)
CCB+	4	165	56	32	0.20, 0.43	Random	Tau^2^ = 0.01; Chi^2^ = 7.26, df = 3 (P = 0.06); I^2^ = 59%	Z = 5.49 (P < 0.00001)
CCB−	4	509	190	34	0.21, 0.47	Random	Tau^2^ = 0.02; Chi^2^ = 28.66, df = 3 (P < 0.00001); I^2^ = 90%	Z = 5.16 (P < 0.00001)
Statins+	6	1236	344	31	0.19, 0.43	Random	Tau^2^ = 0.02; Chi^2^ = 96.14, df = 5 (P < 0.00001); I^2^ = 95%	Z = 5.10 (P < 0.00001)
Statins−	6	181	38	19	0.14, 0.25	Fix	Chi^2^ = 8.00, df = 5 (P = 0.16); I^2^ = 38%	Z = 6.77 (P < 0.00001)
Country								
USA	16	2857	814	33	0.26, 0.40	Random	Tau^2^ = 0.02; Chi^2^ = 258.05, df = 15 (P < 0.00001); I^2^ = 94%	Z = 9.60 (P < 0.00001)
Other countries	4	307	88	27	0.08, 0.46	Random	Tau^2^ = 0.04; Chi^2^ = 54.00, df = 3 (P < 0.00001); I^2^ = 94%	Z = 2.78 (P = 0.005)
							Test for subgroup differences: Chi^2^ = 0.29, df = 1 (P = 0.59), I^2^ = 0%	
Mental stress (MS)								
Public speaking	6	1606	314	22	0.17, 0.28	Random	Tau^2^ = 0.00; Chi^2^ = 34.85, df = 5 (P < 0.00001); I^2^ = 86%	Z = 7.95 (P < 0.00001)
Mental arithmetic	3	354	100	26	0.12, 0.40	Random	Tau^2^ = 0.01; Chi^2^ = 19.63, df = 2 (P < 0.0001); I^2^ = 90%	Z = 8.86 (P < 0.00001)
Anger recall	2	151	52	34	0.27, 0.42	Fix	Chi^2^ = 0.23, df = 1 (P = 0.63); I^2^ = 0%	
Two MS	5	536	196	37	0.23, 0.51	Random	Tau^2^ = 0.02; Chi^2^ = 50.67, df = 4 (P < 0.00001); I^2^ = 92%	Z = 5.12 (P < 0.00001)
Three or more than three MS	4	517	240	43	0.24, 0.61	Random	Tau^2^ = 0.03; Chi^2^ = 51.27, df = 3 (P < 0.00001); I^2^ = 94%	Z = 4.51 (P < 0.00001)
							Test for subgroup differences: Chi^2^ = 11.21, df = 4 (P = 0.02), I^2^ = 64.3%	
Diagnostic techniques								
SPECT	9	1893	417	26	0.20, 0.32	Random	Tau^2^ = 0.01; Chi^2^ = 70.54, df = 8 (P < 0.00001); I^2^ = 89%	Z = 8.68 (P < 0.00001)
RNV	2	177	49	38	− 0.01, 0.78	Random	Tau^2^ = 0.08; Chi^2^ = 22.05, df = 1 (P < 0.00001); I^2^ = 95%	Z = 1.90 (P = 0.06)
ECG	2	146	24	16	0.10, 0.22	Fix	Chi^2^ = 1.03, df = 1 (P = 0.31); I^2^ = 3%	Z = 5.33 (P < 0.00001)
Echocardiography	3	468	198	41	0.21, 0.61	Random	Tau^2^ = 0.03; Chi^2^ = 37.63, df = 2 (P < 0.00001); I^2^ = 95%	Z = 3.97 (P < 0.0001)
Two types of diagnostic technique	4	480	214	43	0.33, 0.54	Random	Tau^2^ = 0.01; Chi^2^ = 16.19, df = 3 (P = 0.001); I^2^ = 81%	Z = 8.15 (P < 0.00001)
							Test for subgroup differences: Chi^2^ = 23.61, df = 4 (P < 0.0001), I^2^ = 83.1%	
Myocardial ischemia measurements								
LVEF decrease	2	220	42	19	0.14, 0.24	Fix	Chi^2^ = 0.02, df = 1 (P = 0.90); I^2^ = 0%	Z = 7.22 (P < 0.00001)
WMA	3	197	99	51	0.34, 0.69	Random	Tau^2^ = 0.02; Chi^2^ = 12.46, df = 2 (P = 0.002); I^2^ = 84%	Z = 5.78 (P < 0.00001)
ST depression	2	146	24	16	0.10, 0.22	Fix	Chi^2^ = 1.03, df = 1 (P = 0.31); I^2^ = 3%	Z = 5.33 (P < 0.00001)
MPD	9	1893	417	26	0.20, 0.32	Random	Tau^2^ = 0.01; Chi^2^ = 70.54, df = 8 (P < 0.00001); I^2^ = 89%	Z = 8.68 (P < 0.00001)
Two or more than two measurements	4	454	216	45	0.37, 0.53	Random	Tau^2^ = 0.01; Chi^2^ = 13.32, df = 3 (P = 0.004); I^2^ = 77%	Z = 11.07 (P < 0.00001)
							Test for subgroup differences: Chi^2^ = 47.23, df = 4 (P < 0.00001), I^2^ = 91.5%	

*MSIMI* mental stress induced myocardial ischemia, *MI* myocardial infarction, *PTCA* percutaneous coronary angioplasty, *CABG* coronary artery bypass graft, *ACEI* angiotensin converting enzyme inhibitor, *ARB* angiotensin receptor block, *CCB* calcium-channel blocker, *MS* mental stress, *SPECT* single photon emission computed tomography, *RNV* radionuclide ventriculography, *ECG* electrocardiography, *VEST* ventricular function monitor, *LVEF* left ventricle ejection fraction, *WMA* wall motion abnormality, *WMA* wall motion abnormality, *MPD* myocardial perfusion defects

### Potential associated factors of MSIMI

#### History of post MI

**Fig. 3:**
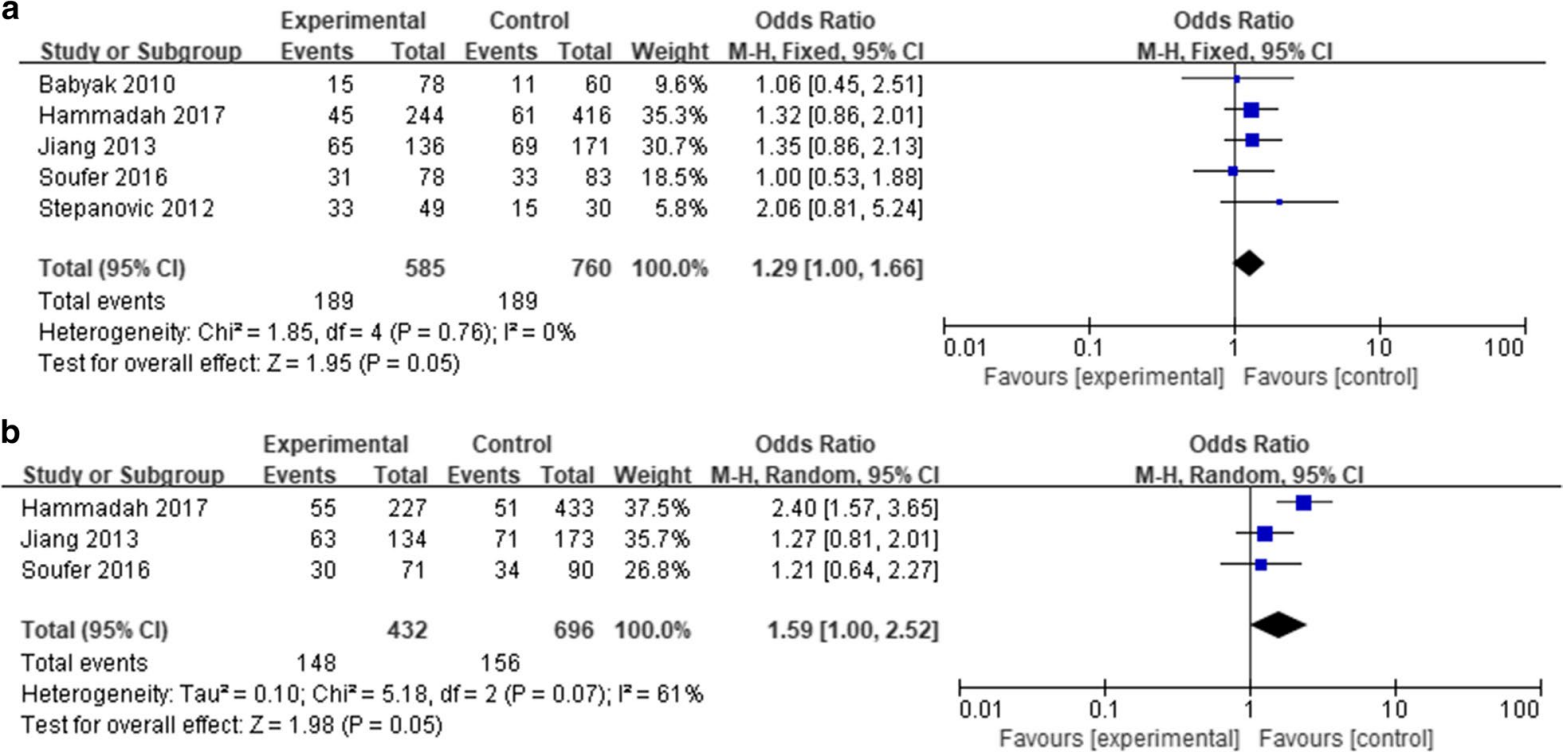
Associated factors of MSIMI; A) The comparison of MSIMI between patients with or without history of post MI; B) The comparison of MSIMI between patients with or without history of CABG

Five articles [19, 22, 25, 31, 33] were selected in the subgroup comparison of post MI history, including 585 patients with post MI and 760 without. Difference of MSIMI was found between patients with post MI and patients without (RR: 1.29, 95% CI 1.00–1.66, P = 0.05). This result indicated that patients with post MI history might be at higher risk of MSIMI (Fig. 3a, Table 4).

**Table 4:** Subgroup comparisons results of the meta-analysis

No.	Comparison	Studies (n)	Sample size	Effect Model	RR/MD/SMD	Heterogeneity	Test for overall effect
1	Female vs. male	11	2131	Fix	1.17 [0.93, 1.48]	Chi^2^ = 14.36, df = 10 (P = 0.16); I^2^ = 30%	Z = 1.35 (P = 0.18)
2	White vs. other races	3	536	Fix	0.75 [0.48, 1.17]	Chi^2^ = 1.32, df = 2 (P = 0.52); I^2^ = 0%	Z = 1.26 (P = 0.21)
3	Smoke+ vs. Smoke−	8	1578	Fix	1.10 [0.86, 1.40]	Chi^2^ = 8.85, df = 7 (P = 0.26); I^2^ = 21%	Z = 0.72 (P = 0.47)
4	Hypertension+ vs. Hypertension−	8	1578	Fix	1.07 [0.80, 1.42]	Chi^2^ = 5.33, df = 7 (P = 0.62); I^2^ = 0%	Z = 0.43 (P = 0.66)
5	Hyperlipidemia+ vs. Hyperlipidemia−	8	1572	Fix	1.13 [0.80, 1.60]	Chi^2^ = 3.67, df = 7 (P = 0.82); I^2^ = 0%	Z = 0.70 (P = 0.48)
6	Diabetes+ vs. Diabetes−	8	1578	Fix	1.26 [0.98, 1.62]	Chi^2^ = 12.07, df = 7 (P = 0.10); I^2^ = 42%	Z = 1.83 (P = 0.07)
7	Depression+ vs. Depression−	2	389	Fix	1.36 [0.78, 2.39]	Chi^2^ = 0.06, df = 1 (P = 0.80); I^2^ = 0%	Z = 1.09 (P = 0.28)
8	Post MI+ vs. Post MI−	5	1345	Fix	1.29 [1.00, 1.66]	Chi^2^ = 1.85, df = 4 (P = 0.76); I^2^ = 0%	Z = 1.95 (P = 0.05)
9	PTCA+ vs. PTCA−	3	1128	Fix	0.88 [0.67, 1.16]	Chi^2^ = 0.53, df = 2 (P = 0.77); I^2^ = 0%	Z = 0.90 (P = 0.37)
10	CABG+ vs. CABG−	3	1128	Random	1.59 [1.00, 2.52]	Tau^2^ = 0.10; Chi^2^ = 5.18, df = 2 (P = 0.07); I^2^ = 61%	Z = 1.98 (P = 0.05)
11	Aspirn+ vs. Aspirin−	5	1279	Fix	0.93 [0.65, 1.34]	Chi^2^ = 0.34, df = 4 (P = 0.99); I^2^ = 0%	Z = 0.36 (P = 0.72)
12	Other antiplatelet agent+ & Other antiplatelet agent−	3	1128	Fix	1.21 [0.91, 1.61]	Chi^2^ = 1.94, df = 2 (P = 0.38); I^2^ = 0%	Z = 1.29 (P = 0.20)
13	ACEI+ vs. ACEI−	5	1279	Fix	1.13 [0.87, 1.46]	Chi^2^ = 2.12, df = 4 (P = 0.71); I^2^ = 0%	Z = 0.92 (P = 0.36)
14	ARB+ vs. ARB−	2	967	Random	1.22 [0.53, 2.82]	Tau^2^ = 0.26; Chi^2^ = 3.53, df = 1 (P = 0.06); I^2^ = 72%	Z = 0.46 (P = 0.64)
15	Beta-block+ vs. Beta-block−	6	1417	Fix	1.05 [0.78, 1.41]	Chi^2^ = 2.50, df = 5 (P = 0.78); I^2^ = 0%	Z = 0.32 (P = 0.75)
16	CCB+ vs. CCB−	4	674	Fix	0.84 [0.58, 1.22]	Chi^2^ = 0.78, df = 3 (P = 0.85); I^2^ = 0%	Z = 0.92 (P = 0.36)
17	Statin+ vs. Statin−	6	1417	Fix	1.18 [0.80, 1.75]	Chi^2^ = 4.30, df = 5 (P = 0.51); I^2^ = 0%	Z = 0.83 (P = 0.40)

*ACEI* angiotensin converting enzyme inhibitor, *ARB* angiotensin receptor block, *CCB* calcium-channel blocker, *MI* myocardial ischemia

#### History of CABG

Three articles [22, 25, 42] were selected in the subgroup comparison of history of CABG, including 432 patients with CABG history and 696 patients without. Difference of MSIMI was found between patients with CABG and patients without (RR: 1.59, 95% CI 1.00–2.52, P = 0.05), indicating that patients with history of CABG might be at higher risk of developing MSIMI (Fig. 3b, Table 4).

#### History of diabetes

Eight articles [19, 20, 22, 25, 28, 29, 31, 33] were selected in the subgroup comparison of history of diabetes, including 608 patients with diabetes history and 1416 patients without. Although no significant difference was found (RR: 1.26, 95% CI 0.98–1.62, P = 0.07), we still considered the potential risk of diabetes in MSIMI due to its impact in coronary artery disease.

#### Other characteristics

Other characteristics were also conducted in this meta-analysis including sex (RR: 1.17, 95% CI 0.93–1.48, P = 0.18), race (RR: 0.75, 95% CI 0.48–1.17, P = 0.21), smoking (RR: 1.10, 95% CI 0.86–1.40, P = 0.47), hypertension (RR: 1.07, 95% CI 0.80–1.42, P = 0.66), hyperlipidemia (RR: 1.13, 95% CI 0.80–1.60, P = 0.48), PTCA (RR: 0.88, 95% CI 0.67–1.16, P = 0.37), depression (RR: 1.36, 95% CI 0.78–2.39, P = 0.28), drug history such as aspirin (RR: 0.93, 95% CI 0.65–1.34, P = 0.72), other antiplatelets (RR: 1.21, 95% CI 0.91–1.61, P = 0.20), ACEI (RR: 1.13, 95% CI 0.87–1.46, P = 0.36), ARB (RR: 1.22, 95% CI 0.53–2.82, P = 0.64), β-block (RR: 1.05, 95% CI 0.78–1.41, P = 0.75), CCB (RR: 0.84, 95% CI 0.58–1.22, P = 0.36), statins (RR: 1.18, 95% CI 0.80–1.75, P = 0.40), and no significant difference was found (Table 4).

### Diagnostic methods of MSIMI

**Fig. 4:**
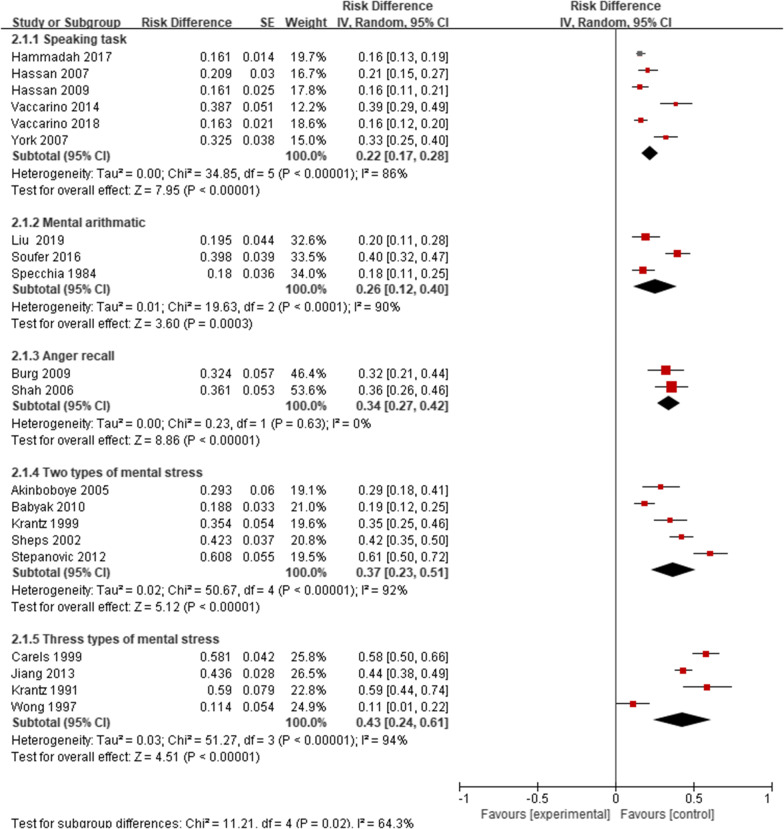
The prevalence of MSIMI by different types of mental stress

**Fig. 5:**
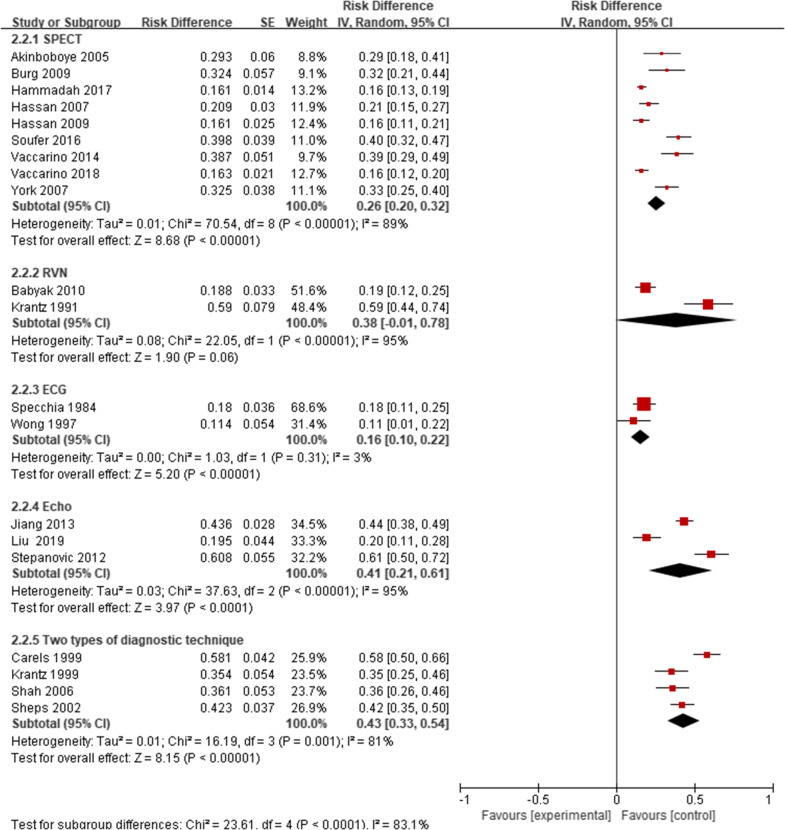
The prevalence of MSIMI by different types of diagnostic techniques

**Fig. 6:**
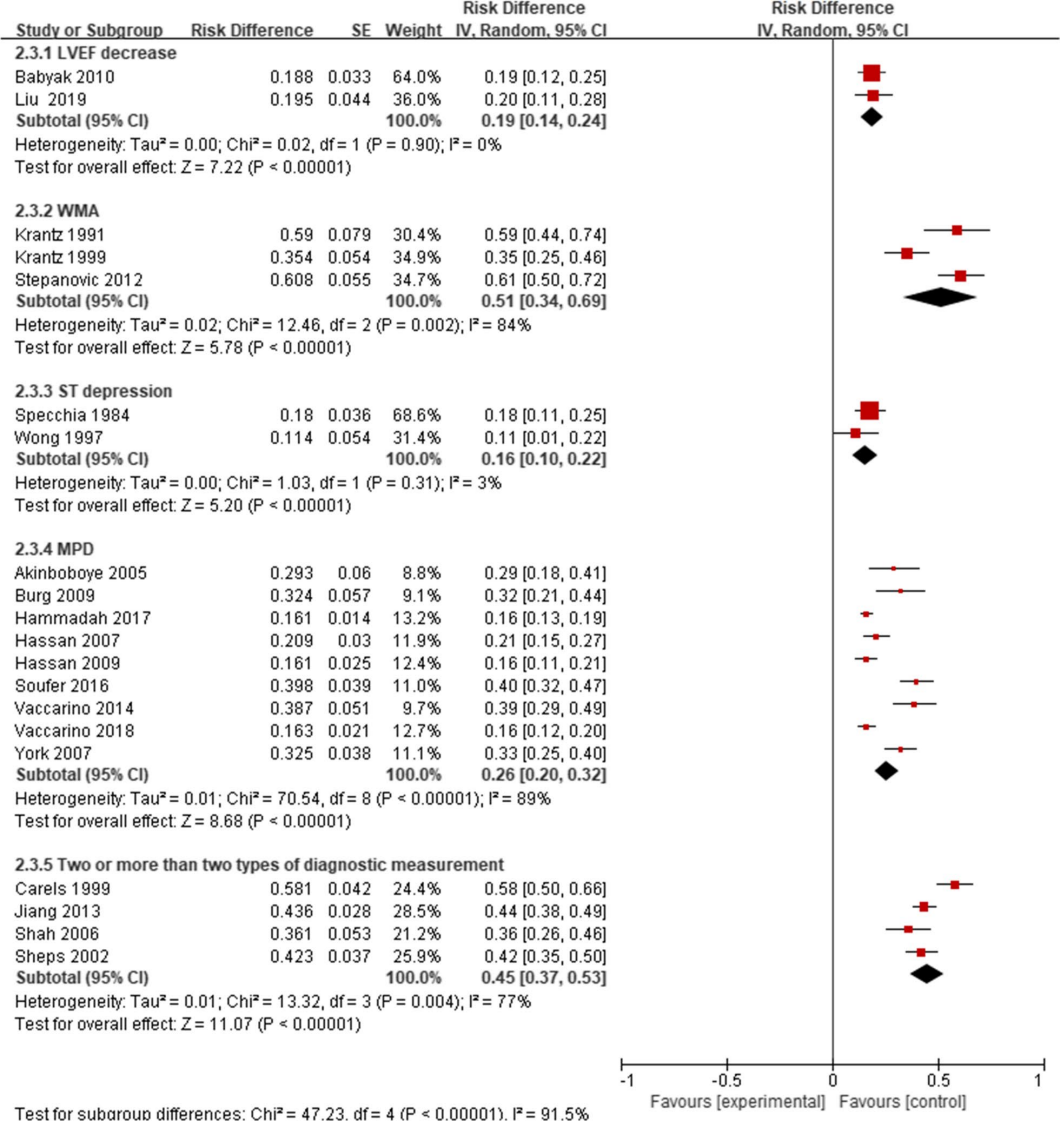
The prevalence of MSIMI by different types of diagnostic measurements

There were significant differences in the prevalence of MSIMI in different types of mental stress, diagnostic techniques, and diagnostic measurements. The prevalence of MSIMI detected by Public Speaking was 22%, Mental arithmetic was 26%, Anger recall was 34%, Two types was 37%, three or more than three types was 43%, and the result was significant (P = 0.02). The results indicated that two and more than two types of mental stress could be more likely to induce MSIMI. The prevalence of MSIMI detected by different types of diagnostic techniques and diagnostic measurements showed significant difference (Table 3, Figs. 4, 5, 6).

Meta-regression was performed to identify the potential moderators in the prevalence of MSIMI, including publication date, sample size, country, different types of mental stress, different types of diagnostic techniques, and different types of myocardial ischemia measurements (Tables 3 and 5).

**Table 5:** Univariate and multivariate meta-regression analyses of potential sources of heterogeneity

Heterogeneity Factors	exp(b)	SE	t	P	95% CI	tau^2^	I-squared_res	Adj R-squared
Univariate								
Publication year	0.9977	0.0038	− 0.61	0.549	0.9896, 1.0058	0.0216	93.69%	− 4.07%
Sample size	0.9996	0.0002	− 1.29	0.215	0.9992, 1.0002	0.0200	92.70%	3.56%
Country	0.9433	0.0815	− 0.68	0.508	0.7867, 1.1311	0.0214	94.23%	− 3.41%
Mental stress	1.0508	0.0201	2.59	0.018	1.0094, 1.0938	0.0151	88.73%	26.96%
Diagnostic techniques	1.0395	0.0200	2.01	0.060	0.9983, 1.0825	0.017	89.82%	17.71%
Diagnostic measurements	1.0187	0.0282	0.67	0.512	0.9611, 1.0797	0.0212	93.95%	− 2.46%
Multivariate						0.0151	87.62%	26.97%
Mental stress	1.0433	0.0260	1.70	0.108	0.9896, 1.0999			
Diagnostic techniques	1.0145	0.0239	0.61	0.551	0.9650, 1.0665			
Diagnostic measurements	1.0263	0.0250	1.07	0.302	0.9747, 1.0807			

On univariate meta-regression, types of mental stress (exp(b): 1.0508, SE: 0.0201, P: 0.018) were associated with the prevalence of MSIMI, while no significance was found in other factors including publication year (exp(b): 0.9977, SE: 0.0038, P: 0.549), sample size (exp(b): 0.9996, SE: 0.0002, P: 0.215), country (exp(b): 0.9433, SE: 0.0815, P: 0.508), diagnostic techniques (exp(b): 1.0395, SE: 0.0200, P: 0.060) and diagnostic measurements (exp(b): 1.0187, SE: 0.0282, P: 0.512). Therefore, different types of mental stress might contribute to the prevalence of MSIMI.

Multivariate meta-regression was performed including mental stress, diagnostic technique, and diagnostic criteria. No significant difference was found: mental stress (exp(b): 1.0433, SE: 0.0260, P: 0.108), diagnostic techniques (exp(b): 1.0145, SE: 0.0239, P: 0.551), diagnostic measurements (exp(b): 1.0263, SE: 0.0250, P: 0.302) (Table 5).

Our results indicated that different types of mental stress might influence the prevalence of MSIMI in CAD patients.

### Comparisons of different diagnostic techniques

In all twenty selected articles, there were four articles indicating that different diagnostic techniques might lead to different prevalence of MSIMI in the same population. Two articles compared SPECT and PAT (peripheral arterial tonometry) which was not recognized as a standard criterion. In Burg’s study, the prevalence of MSIMI was 32.35% by SPECT, and 42.65% by PAT, while only 19.12% by both. The area under the curve (AUC) was 0.613 (SE, 0.065, one-sided P = 0.04). In Hassan’s study, when comparing SPECT and PAT, the area under the curve (AUC) was 0.59 (95% CI 0.48–0.69, P = 0.116). In addition, Carels’ study showed that the prevalence of MSIMI was 33.09% by RNV, and 44.12% by ambulatory ECG, while only 19.2% by both. Krantz’s study showed that the prevalence of MSIMI was 55.7% by RNV, and 57% by echocardiography (Table 6).

**Table 6:** Comparisons of different diagnostic techniques

No.	First author	Publication data	Sample size	Mental stress	First DT	MSIMI	Second DT	MSIMI	Combined techniques	ROC curve
1	Burg	2009	68	Ager recall	SPECT	22 (32.35%)	PAT	29 (42.65%)	13 (19.12%)	AUC: 0.613 (SE, 0.065, one-sided P = 0.04)
2	Carels	1999	136	Mental arithmetic Public speaking Mirror tracing Reading Type A structured interview	RNV	45 (33.09%)	Ambulatory ECG	60 (44.12%)	26 (19.2%)	–
3	Hassan	2009	211	Public speaking	SPECT	34 (16.11%)	PAT	–	–	AUC: 0.59; 95% CI 0.48–0.69, P = 0.116
4	Krantz	1999	79	Mental arithmetic public speech	RNV	44 (55.7%)	Echocardiography	45 (57%)	–	–

*ECG* electrocardiogram, *SPECT* single photon emission computed tomography, *RNV* radionuclide ventriculography, *PAT* peripheral arterial tonometry

### Sensitivity analysis

**Fig. 7:**
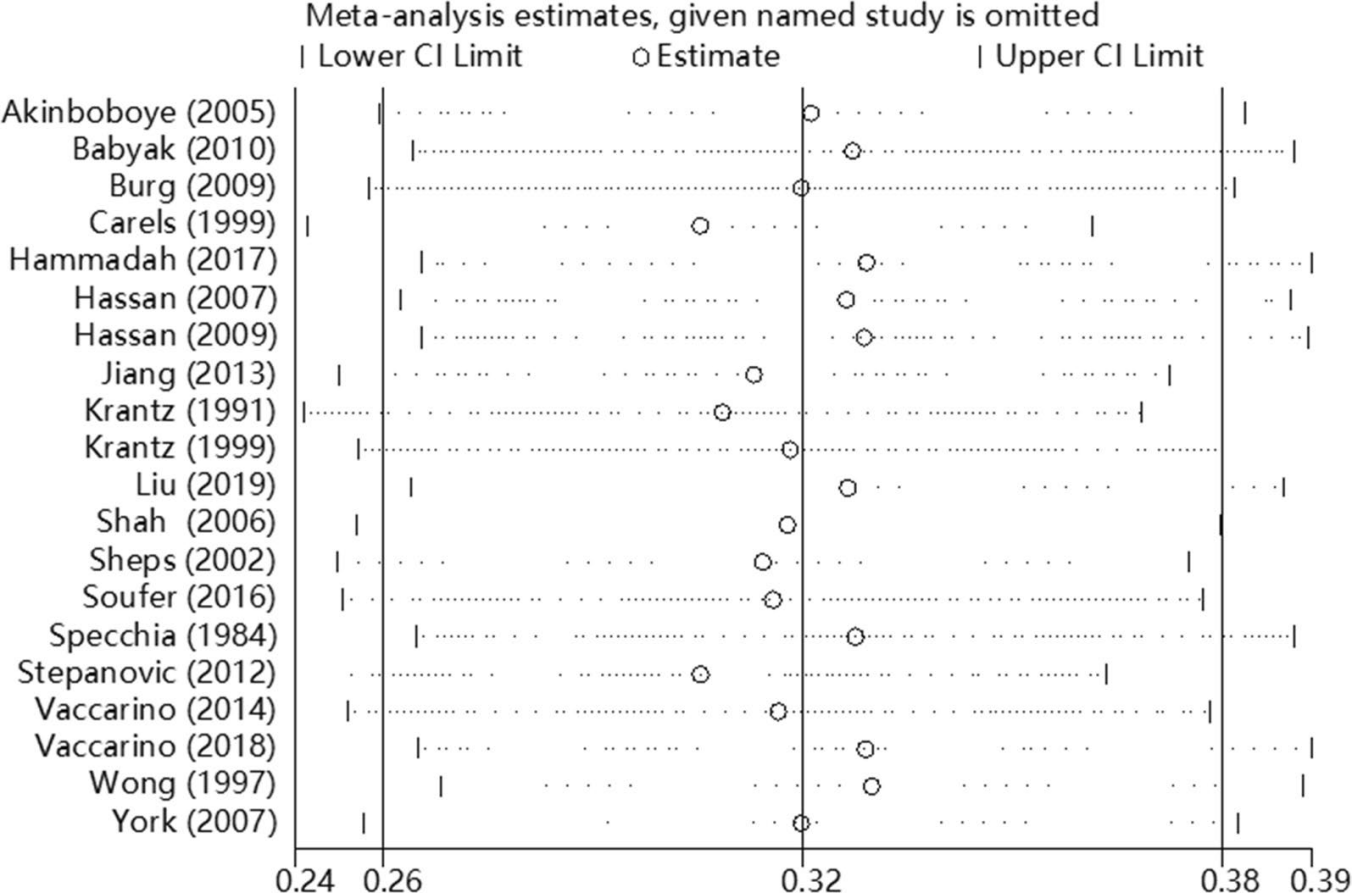
Sensitivity analysis on this meta-analysis

We performed sensitivity analysis by Stata 12.0, excluding a single study each time to detect the influence of individual dataset on pooled ESs. The results demonstrated that no significant change was found after omitting any of the study (Fig. 7).

### Publication bias

**Fig. 8:**
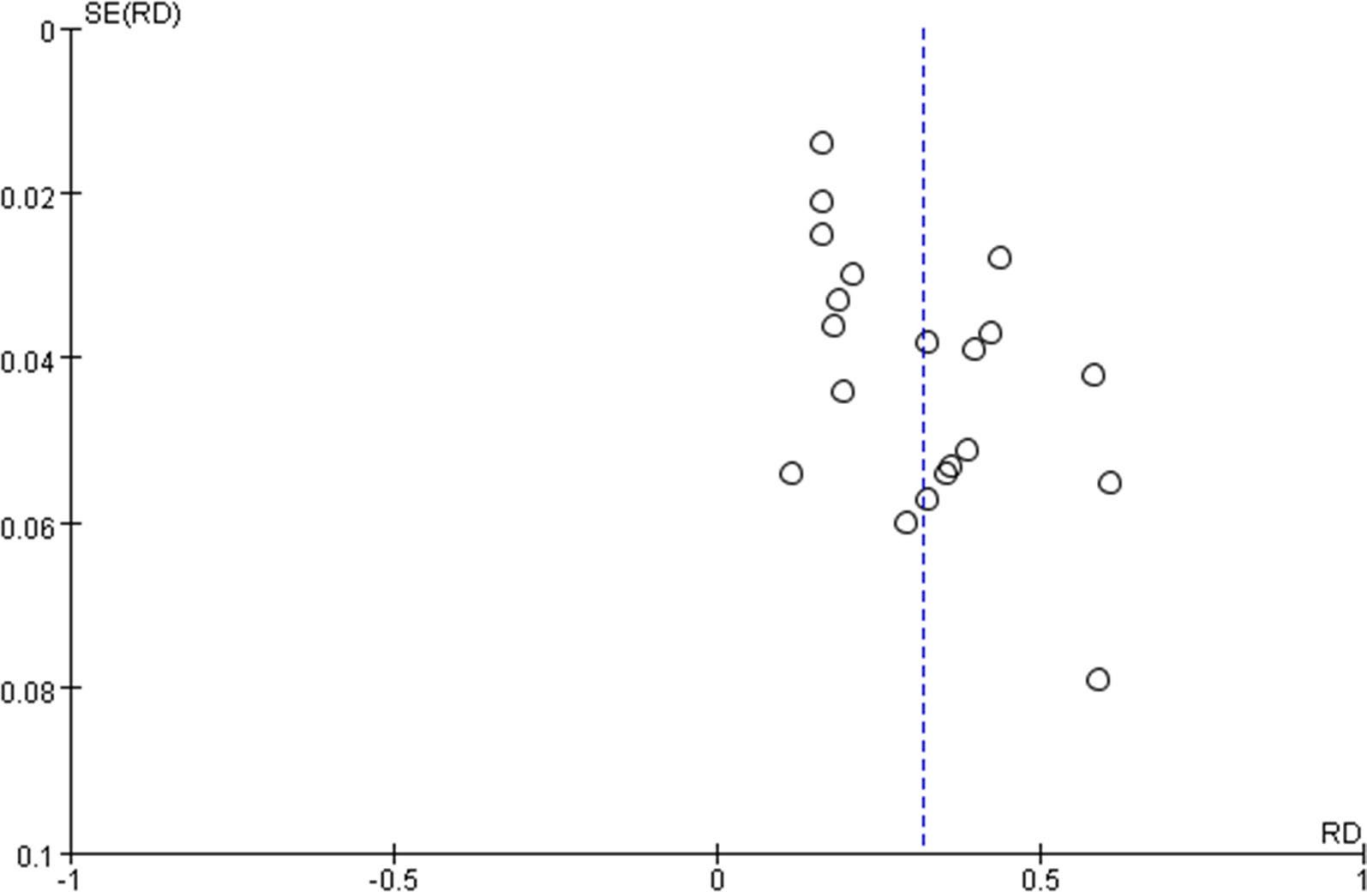
Funnel plot of this meta-analysis

Publication bias was detected by funnel plot and modified Begg’ test. The funnel plot was symmetric and the Begg’ test presented no significant publication bias in this meta-analysis (Z = 1.69, P > 0.05) (Fig. 8).

## Discussion

In this meta-analysis, the pooled estimated prevalence of MSIMI in CAD patients was as high as 32%. Consequently, it attracted our attentions to summarize the related factors and diagnostic methods of MSIMI in CAD patients to better understand the MSIMI assessment. To the best of our knowledge, this is the first meta-analysis concentrated on this topic.

### Associated factors of MSIMI

Subgroups analyses elucidated that CAD patients with history of diabetes, or post MI, or CABG might be associated with a higher risk of MSIMI, though the statistical analysis was not significant enough. Diabetes is considered as a risk factor of CAD, due to the dysfunction of micro- and macro- vascular damaged by hyperglycemia [38] via inflammation pathway. The sudden mental stress results in the lack of blood flow and oxygen, and thus causes myocardial ischemia [9]. Patients with diabetes, or post MI, or CABG, have worse cardiac conditions because of existing cardiac cell damage and microvascular dysfunction. Therefore, they are more vulnerable to myocardial ischemia when mental stress occurs.

Our results indicated that there was no significant difference of developing MSIMI between females and males, and between patients with depression and without. The results in this meta-analysis were different from some individual studies.

Vaccarino et al. [35] elucidated that young women with CHD were more likely to develop MSIMI, which was almost fourfold higher than men. Another study of Vaccarino reported similar conclusions that mechanisms in MSIMI could be different in females and males, and the higher morbidity of MSIMI in females might be related with the microcirculatory dysfunction. Samad et al. [39] suggested that the higher morbidity of MSIMI in females might be associated with platelet activity. To our surprise, our results in the present study did not suggest sex as a significant risk factor. This inconsistency might be due to: (1) the different samples and proportion of females and males in each study; (2) the studies were from different regions. More original researches should be done to further study the relationship between sex and MSIMI.

Depression is an independent risk factor of cardiovascular diseases [40]. Jiang et al. [7] suggested that patients with mild to moderate depressive symptoms were at higher risk of MSIMI. In this research, depression was assessed by Center for Epidemiological Studies-Depression scale (CES-D). However, only four articles mentioning depression were included in the present meta-analysis, and no significant importance was found in depression as a risk factor for MSIMI.

In addition, anger [5], sever left ventricular dysfunction, and anxiety [6] have been considered as severe factors in MSIMI, but the evidence is not enough.

### Diagnostic methods of MSIMI

We found significant differences in MSIMI prevalence detected by different mental stress, diagnostic techniques and diagnostic measurement. Univariate meta-regression elucidated the potential link between types of mental stress and MSIMI. We postulated some potential reasons for this association. First, the activation of different signal pathway may lead to different consequences. The mechanism of MSIMI involves the strong interaction between heart and the brain. Mental stress can activate hypothalamic pituitary adrenocortical axis, sympathetic nervous system, adrenomedullar hormonal system, and parasympathemic nervous system via releasing different hormones or neurotransmitters which can have different impact. Second, individual differences may play an important role. In Table 1, we described the types of mental stress in all the included studies. The common types involve mental arithmetic, anger call, public speech, mirror trace, Stroop color-word test et al. We found that the prevalence of MSIMI induced by one type of mental stress was 22–34%, two types of mental stress was 37%, and three types yield 43% (Table 3). According to our own clinical observations, trace mirror seemed to be a pleasure rather than emotional stress for those who are good at designing or drawing, while mental arithmetic could be a serious stress to them for most of them are afraid of mathematics; vise verse for those who are skilled at mental arithmetic. The phenomenon implied that we should consider individual differences in the consequence caused by different types of mental stress task, which is consistent with Bremner et al’s study. Bremner [41] conducted a study with the intent of revealing the association between brain and MSIMI. It was found that mental arithmetic was associated with left insula activation, while public speaking was associated with right pre/post-central gyrus and middle temporal gyrus activation. In the context of MSIMI, different types of mental stress might active or deactivate different brain regions, which would promote or inhibit cardiac responses. Therefore, we suggest that researchers should consider individual differences in different types of mental stress task while assessing MSIMI, and make a standard together. In our opinion, two different types of mental stress tasks would be better to diagnose MSIMI for the reason that one type might not be eligible to provoke MSIMI, while more than two types might be time and economic consuming.

In addition, we took diagnostic techniques as a pivotal factor in diagnosing MSIMI. As Table 3 showed that the prevalence of MSIMI diagnosed by SPECT was 26%, ECG yielded 16%, while echocardiography yielded 41%, RNV yields 38%. SPECT is a direct way to observe myocardial ischemia via myocardial perfusion defects, demonstrating its vital role in diagnosing MSIMI. Good reproducibility of SPECT has also been identified [42]. However, some patients with MSIMI assessed by echocardiography might be missed.

ECG is a convenient technique, but it is been proved not sensitive enough for MSIMI [36]. Jiang et al. [25] investigated both ECG and echocardiography in distinguishing MSIMI, while no myocardial ischemia was discovered by ECG. Therefore, the false negative of ECG presented low prevalence of MSIMI.

Echocardiography is economical and practical in clinical practice, which could detect LVEF response and wall motion during mental stress simultaneously. Though LVEF decrease could result from myocardial ischemia induced by mental stress and also be consistent with SPECT [43], LVEF response is also influenced by hemodynamics and the basic left ventricular function [18]. Therefore, echocardiography is likely to generate false positive results.

Peripheral arterial tonometry (PAT) is applied to assess microcirculation dysfunction, which is expected to detect myocardial ischemia induced by mental stress. CAD patients with MSIMI have lower PAT ratio according to the studies comparing SPECT and PAT. Some researchers suggested that PAT might have similar detection efficiency compared to SPECT and RNV [20, 44], and more researches remain to further explore the potential role of PAT in detecting MSIMI and make it standardized.

Additionally, increasing researches have been focused on biomarkers that are convenient to achieve and assess, such as neurotransmitters (e.g. epinephrine, norepinephrine [45]), blood coagulation factors (e.g. fibrinogen [46]), cardiac biomarkers (e.g. cTnI [11], cTnT [46]), and inflammatory factors (e.g. IL-6 [47], CRP [29]). These biomarkers are considered to the mechanisms of MSIMI. Consequently, there is bright future in discovering biomarkers for developing economic diagnostic methods of MSIMI.

## Conclusions

In conclusion, the pooled prevalence of MSIMI in CAD patients is 32%. The present meta-analysis implicates that patients with diabetes, or post MI or CABG are more vulnerable to develop MSIMI and different types of mental stress and diagnostic techniques might influence the prevalence of MSIMI. Therefore, it is necessary to formulate a standard diagnostic method for MSIMI, which should be adequate, assessable, and affordable all around world.

## Data Availability

Data and materials will be provided to those who are interested in this meta-analysis by the correspondence.

## Abbreviations

MSIMI: Mental stress induced myocardial ischemia
CAD: Coronary artery diseases
MI: Myocardial infarction
LVEF: Left ventricular ejection fraction
WMA: Wall motion abnormality
ECG: Electrocardiogram
RNV: Radionuclide ventriculography
PTCA: Percutaneous coronary angioplasty
CABG: Coronary artery bypass graft
ACEI: Angiotensin converting enzyme inhibitor
ARB: Angiotensin receptor block
CCB: Calcium-channel blocker
CVD: Cardiovascular diseases
SPECT: Single photonemission computed tomography
MPD: Myocardial perfusion defects
PAT: Peripheral arterial tonometry
CNKI: China National Knowledge Infrastructure
NOS: Newcastle–Ottawa Scale
CES-D: Center for Epidemiological Studies-Depression scale
HPA: Hypothalamic pituitary adrenal
